# Clinical outcomes and risk factors of progressive pulmonary fibrosis in primary Sjögren’s syndrome-associated interstitial lung disease

**DOI:** 10.1186/s12890-023-02562-w

**Published:** 2023-07-19

**Authors:** Yu-Hsuan Chen, Tai-Ju Lee, Hsin-Jung Hsieh, Song-Chou Hsieh, Hao-Chien Wang, Yeun-Chung Chang, Chong-Jen Yu, Jung-Yien Chien

**Affiliations:** 1 Department of Internal Medicine, National Taiwan University Hospital Yunlin Branch, Yunlin, Taiwan; 2 Department of Internal Medicine, National Taiwan University Hospital Hsinchu Branch, Hsinchu, Taiwan; 3 Department of Internal Medicine, National Taiwan University Hospital Jinshan Branch, New-Taipei, Taiwan; 4 Department of Internal Medicine, National Taiwan University Hospital, Taipei, Taiwan; 5 Department of Medical Imaging, National Taiwan University Hospital, Taipei, Taiwan; 6 Division of Chest Medicine, Department of Internal Medicine, National Taiwan University Hospital, No. 7, Zhongshan S. Rd., Zhongzheng Dist., Taipei 100, Taipei, Taiwan

**Keywords:** Progressive fibrosing, Primary Sjögren’s syndrome-associated interstitial lung disease

## Abstract

**Background:**

To investigate the clinical outcomes and risk factors associated with progressive fibrosing interstitial lung disease (PF-ILD) in patients with primary Sjögren’s syndrome-associated interstitial lung disease (pSjS-ILD).

**Methods:**

During 2015–2021, pSjS patients with ILD were retrospectively identified. Patients were grouped into non-PF-ILD and PF-ILD. Demographics, laboratory data, pulmonary function tests (PFTs), images, survival outcomes were compared between groups.

**Results:**

153 patients with SjS-ILD were reviewed, of whom 68 having primary SjS-ILD (pSjS-ILD) were classified into non-PF-ILD (n = 34) and PF-ILD groups (n = 34). PF-ILD group had persistently lower albumin levels and a smaller decline in immunoglobulin G (IgG) levels at the 3rd month of follow-up. The multivariate logistic regression analysis revealed that persistently low albumin levels were associated with PF-ILD. At the 12th month, the PF-ILD group experienced a smaller increase in FVC and a greater decline in the diffusion capacity of carbon monoxide (DLCO) than at baseline. The 3-year overall survival rate was 91.2%, and PF-ILD group had significantly poorer 3-year overall survival rate than non-PF-ILD group (82.4% vs. 100%, p = 0.011). Poor survival was also observed among female patients with PF-ILD.

**Conclusions:**

Among patients with pSjS-ILD, the PF-ILD group had poorer 3-year survival outcomes. Persistent lower albumin level might be the risk factor of PF-ILD. Early lung function tests could be helpful for the early detection of PF-ILD.

## Introduction

Interstitial lung disease (ILD) is a heterogeneous disorder characterized by inflammation and fibrosis of the lung parenchyma [1, 2]. Idiopathic pulmonary fibrosis (IPF) is a prototype of progressive fibrosing ILD with poor prognosis. However, other ILD subtypes, such as idiopathic nonspecific interstitial pneumonia (iNSIP), fibrotic hypersensitivity pneumonitis (HP), and connective tissue disease-associated ILDs (CTD-ILDs), also develop progressive pulmonary fibrosis with worsening symptoms, decline in lung function, increased extent of fibrosis on high-resolution computed tomography (HRCT), and early mortality [1–5]. The definition of progressive fibrosing ILD (PF-ILD) varies between studies. Many studies followed the criteria of disease progression based on the INBUILD study [6], which was the first and largest phase 3 clinical trial to explore the therapeutic effect of anti-fibrotic agents in patients with PF-ILD. Disease progression in INBUILD study was defined as a relative decline in forced vital capacity (FVC) ≧ 10% of predicted values, a relative decline in FVC ≧ 5–10% of predicted values with worsening respiratory symptoms or increased fibrosis extent on HRCT, or worsening respiratory symptoms with an increased extent of fibrosis in previous 24 months.

Approximately 30% of non-IPF ILD cases develop PF-ILD [2, 3]. This proportion varies according to the underlying pathology. Among the CTD-ILDs, the proportions of PF-ILD in patients with rheumatoid arthritis-ILD (RA-ILD), systemic sclerosis-ILD (SSc-ILD), and primary Sjögren’s syndrome-associated ILD (pSjS-ILD) were approximately 34.5%, 33.3%, and 21.7%, respectively [1]. To the best of our knowledge, the risk factors for developing PF-ILD in RA-ILD and SSc-ILD patients have been well investigated [7–9], but not in patients with SjS-ILD. Therefore, we aimed to identify prevalence, clinical outcomes and risk factors of PF-ILD in patients with pSjS-ILD.

## Materials and methods

This retrospective study was approved by the Institutional Review Board of National Taiwan University Hospital (202111037RINB). Information for this study was collected from the Integrated Medical Database of the National Taiwan University Hospital between January 2015 and August 2021.

First, adult patients with a diagnosis coding number of M35.02 (Sjögren syndrome with lung involvement) based on the coding system of the International Classification of Diseases, 10th Revision, Clinical Modification (ICD-10-CM) and HRCT images were available for interpretation. Second, the HRCT images of these patients were independently reviewed by three pulmonologists, and patients with ILD were identified [10]. Thirdly, PF-ILD was defined as a relative decline in FVC ≧ 10% of predicted values, a relative decline in FVC ≧ 5–10% of predicted values with worsening of respiratory symptoms or increased fibrosis extent on HRCT, or worsening of symptoms and imaging in previous 24 months. Therefore, patients with no baseline pulmonary function test (PFT), no PFT or chest images in the previous 24 months, or death within 6 months (for the exclusion of rapid progression-ILD) were excluded. Finally, the electronic medical records (EMR) of patients with Sjögren’s syndrome with interstitial lung disease (SjS-ILD) were reviewed in detail by one rheumatologist, and the patients were further confirmed to have primary Sjögren’s syndrome (pSjS) using the 2002 American-European Consensus Group (AECG) criteria or the 2016 American College of Rheumatology and European League Against Rheumatism (ACR/EULAR) Classification Criteria for Primary Sjögren’s syndrome [11, 12].

Detailed demographic and clinical features, including age, sex, smoking history, hypertension, diabetes mellitus, and HRCT patterns, were recorded until the last follow-up appointment or at the end of the 3-year follow-up period. The date of the first abnormal HRCT examination was defined as the date of ILD diagnosis (baseline). Total fibrosis score (TFS), a CT scoring method, was used to quantify fibrosis extent [13]. The higher the score of TFS, the greater the extent of fibrosis. Chest images, including HRCT patterns and TFS, were reviewed at the baseline and during the 24- month follow-up period. Results of pulmonary function tests results were recorded at baseline, the 12th month and the 24th month. Laboratory results were extracted, including complete blood cell counts, autoimmune profiles, and inflammatory markers at baseline and the 3rd months. The proportions of enrolled patients using corticosteroids and conventional and biological disease-modifying antirheumatic drugs (DMARDs) were recorded. We also collected data on the prevalence of PF-ILD, 3-year overall survival and all-cause mortality.

### Statistical analysis

Statistical analyses were performed using the IBM SPSS Statistics version 26. Continuous variables, reported as medians with interquartile ranges, were analyzed using the Mann–Whitney U test, as appropriate. Categorical variables, presented as absolute numbers and frequencies (percentages), were assessed using the chi-square test. Logistic regression analysis was used to determine any associations between parameters. Overall survival was defined as the time from the date of ILD diagnosis to the date of death due to any cause, or the end of the 3-year follow-up period. Survival estimates were performed using the Kaplan–Meier method for the overall population. Statistical significance was set at p < 0.05. significant.

## Results

A total of 283 adult patients whose ICD-10-CM number was M35.02 and HRCT images were available for interpretation were enrolled. Second, the HRCT scans of 283 patients were reviewed, and only 153 patients were confirmed to have Sjögren syndrome with interstitial lung disease (SjS-ILD). Third, 51 patients who had no baseline PFT (n = 30),, no PFT or chest images in the previous 24 months (n = 16), or death within 6 months (n = 5) were excluded. Lastly, the EMR of the remaining 102 patients were reviewed, and 68 patients were identified as having pSjS using the 2002 AECG criteria or 2016 ACR/EULAR classification criteria. The time of ILD diagnosis was defined as the date of the first HRCT examination, which also served as the baseline. The total observation period was 3 years after ILD diagnosis.

**Fig. 1:**
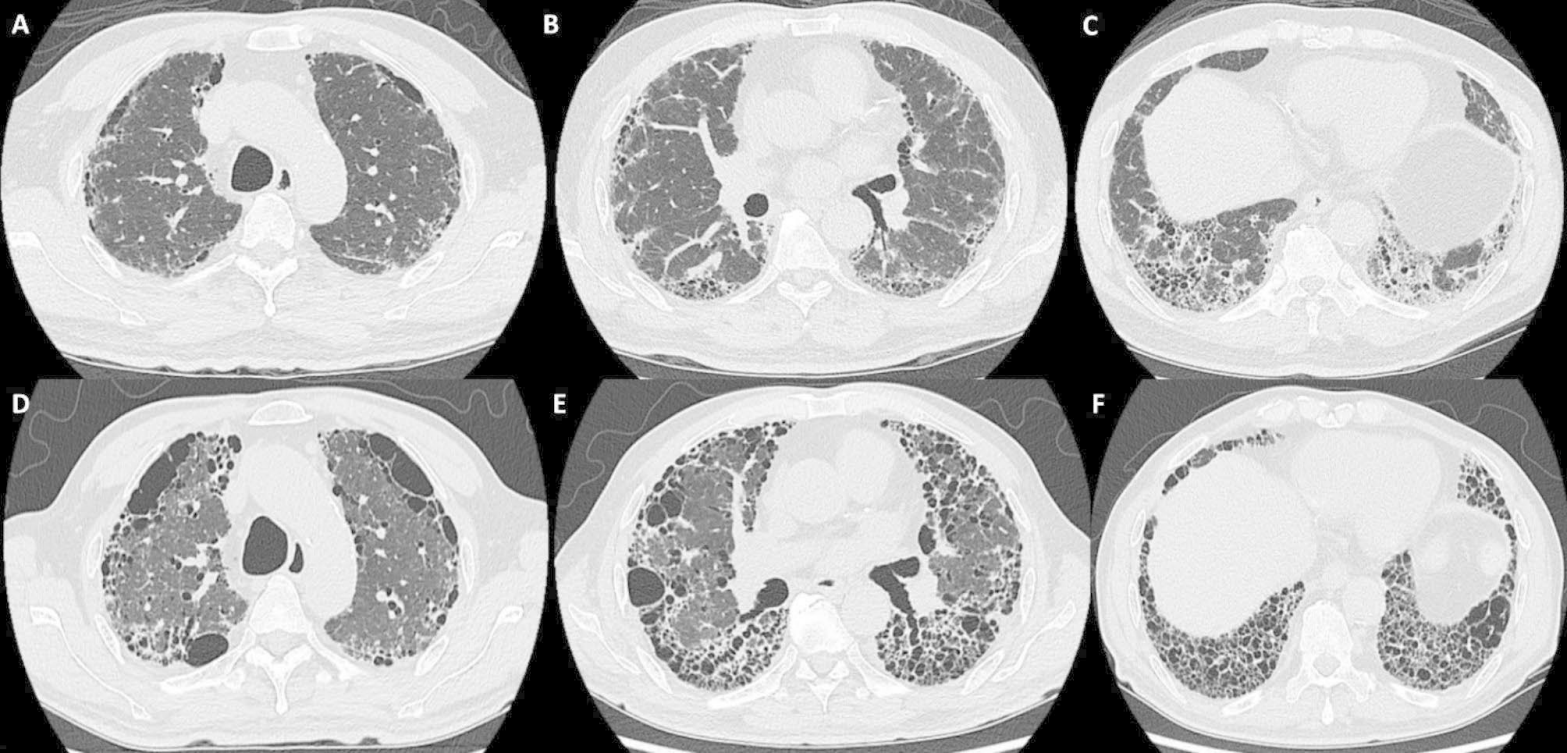
High resolution computed tomography imaging of the chest in a patient with Sjögren’s syndrome-associated interstitial lung disease (PF-ILD). Serial axial images at baseline (Panel **A-C**) and at 24 months (Panel **D-F**)

The prevalence of PF-ILD in pSjS-ILD patients was 50% (34/68)(Fig. 1), and the remaining 50% (34/68) were considered to be stable or to have improvement, namely non-PF-ILD group. There were no significant differences in the demographics, pulmonary function tests, hemograms, autoimmune profiles, inflammatory markers, and HRCT patterns between the PF-ILD and non-PF-ILD groups in the baseline characteristics (Table 1). The PF-ILD diagnosis was most commonly established based on the presence of a relative FVC decline < 5% with both worsening of symptoms and chest imaging (27 PF-ILD patients, 79%). There were 5 patients (14%) classified as PF-ILD using the criterion of a relative FVC decline ≧ 10% of predicted value.

**Table 1:** Comparison of baseline clinical characteristics between patients with progressive fibrosing interstitial lung disease (PF-ILD) or non-PF-ILD

	Non-PF-ILD (n = 34)	PF-ILD (n = 34)	p value
Age (years)	62.3 (52.1–69.8)	65.1 (53.5–72.6)	0.303
Male	14 (41.2%)	14 (41.2%)	1.000
Smoker	6 (17.6%)	10 (29.4%)	0.392
Hypertension	13 (38.2%)	12 (35.3%)	1.000
Diabetes mellitus	6 (17.6%)	7 (20.6%)	1.000
**Pulmonary function tests**			
FVC (% predicted)	74.45 (60.32–88.02)	80.6 (65.6–89.1)	0.238
DLCO (% predicted)	55.5 (45.6–68.9)	58.05 (42.98–77.25)	0.689
cDLCO (%predicted)	56.5 (45.81–77.53)	59.55 (43.04–76.65)	0.901
FEV1 (% predicted)	76.35 (64.05–91.27)	84.9 (68.2–98.8)	0.172
FEV1/FVC (%)	86.2 (79.2–88.2)	85.3 (80.5–90.4)	0.990
**Hemogram**			
WBC (k/µL)	8.72 (6.04–12.74)	6.99 (5.64–9.26)	0.306
RBC (k/µL)	4.47 (4.01–4.84)	4.25 (3.83–4.80)	0.206
Hemoglobin (g/dL)	13.05 (11.47–14.10)	12.6 (10.7–13.7)	0.294
Platelet (k/µL)	238.5 (198.5-299.2)	262.5 (199.5-332.7)	0.577
Segment (%)	73.05 (66.97–80.30)	70.1 (55.5–81.9)	0.396
Eosinophil (%)	0.9 (0.4–2.7)	2.0 (0.8-3.0)	0.254
Basophil (%)	0.2 (0.1–0.5)	0.3 (0.2–0.6)	0.442
Monocyte (%)	5.25 (4.52-6.00)	5.8 (4.0-7.5)	0.344
Lymphocyte (%)	17.8 (14.7–27.1)	17.6 (10.7–32.1)	0.802
**Autoimmune and inflammatory markers**			
Anti-SSA (U/mL)	197.47 (82.37–240.0)	195.8 (50.4–240.0)	0.494
Anti-SSB (U/mL)	2.76 (0.30-29.75)	0.43 (0.30-20.75)	0.540
ANA positivity	13 (40.6%)	15 (50.0%)	0.610
Anti-ENA positivity	24 (88.9%)	21 (84.0%)	0.698
Anti-dsDNA positivity	2 (8.0%)	5 (16.7%)	0.436
RF (IU/mL)	10.4 (9.8–15.8)	10.4 (9.8–35.6)	0.784
IgG (mg/dL)	1680 (1395–2215)	1520 (1145–2110)	0.180
IgA (mg/dL)	331 (248–450)	335.0 (198.5-610.5)	0.885
IgM (mg/dL)	121.0 (14.4–414.0)	113.0 (72.7–160.0)	0.606
C3 (mg/dL)	113.5 (98.8–132.0)	102.0 (93.7-132.5)	0.341
C4 (mg/dL)	22.0 (18.2–25.5)	22.2 (16.1–27.7)	0.995
CRP (mg/dL)	0.45 (0.12–3.37)	0.73 (0.14–2.63)	0.647
ESR (mm/hr)	32.0 (51.5–17.0)	55 (20–71)	0.132
Albumin (g/dL)	3.9 (3.4–4.2)	3.8 (3.1–4.1)	0.497
Ferritin (ng/mL)	252.9 (117.0-458.5)	414.0 (105.8–786.0)	0.229
D dimer (µg/mL)	0.61 (0.31–1.66)	1.06 (0.43–2.03)	0.240
**High-resolution computed tomography**			
Total fibrosis score	31.6 (18.5–43.9)	24.1 (15.8–42.7)	0.311
Honeycombing	11 (32.4%)	14 (41.2%)	0.615
Reticulation	33 (97.1%)	30 (88.2%)	0.356
Traction bronchiectasis	34 (100%)	32 (94.1%)	0.493
GGO + Traction bronchiectasis	25 (73.5%)	17 (50%)	0.080
Consolidation	9 (26.5%)	10 (29.4%)	1.000
Cyst	3 (8.8%)	8 (23.5%)	0.186
UIP	13 (38.2%)	14 (41.2%)	1.000
NSIP	10 (29.4%)	7 (20.6%)	0.576
LIP	2 (5.9%)	6 (17.6%)	0.259
OP	4 (11.8%)	3 (8.8%)	1.000

Data are presented as n (column %), mean ± SD or median (interquartile range), unless otherwise stated

Abbreviations: ANA: antinuclear; anti-ENA: anti-extractable nuclear antigen; C3/C4: complement component 3/4; CRP: c reactive protein; DLCO: diffusion capacity of carbon monoxide; cDLCO: diffusion capacity of carbon monoxide corrected for hemoglobin; DMARDs: disease-modifying anti-rheumatic drugs; ESR: erythrocyte sedimentation rate; FCV: forced vital capacity; FEV1: forced expiratory volume in 1 s; GGO: ground glass opacity; IgG/A/M: immunoglobulin G/A/M; LIP: lymphocytic interstitial pneumonia; NSIP: nonspecific interstitial pneumonia; OP: organizing pneumonia; PF-ILD: progressive fibrosis-interstitial lung disease; RBC: red blood cell counts; RF: rheumatoid factor; UIP: usual interstitial pneumonia; WBC: white blood cell counts

**Fig. 2:**
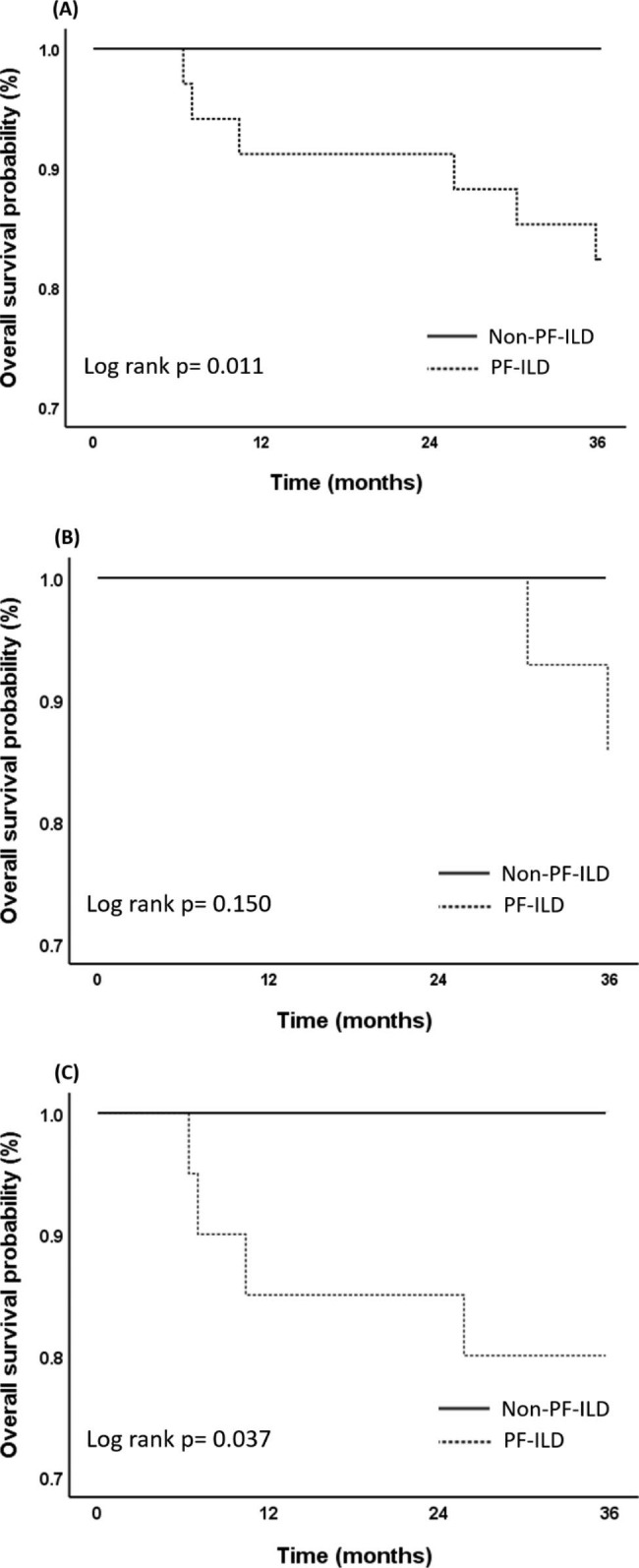
Kaplan-Meier survival analysis for primary Sjögren’s syndrome-associated interstitial lung disease (PF-ILD) (Panel **A**) in male (Panel **B**) and female patients (Panel **C**)

Immunologically, compared to the non-PF-ILD group, lower albumin level (non-PF-ILD vs. PF-ILD: 4.2 vs. 3.85 g/dL, p = 0.025) and less decline in IgG level at the 3rd month of follow-up (non-PF-ILD vs. PF-ILD: -14% vs. 0%, p = 0.026) were found in PF-ILD group with statistical significance (Table 2). The proportions of patients treated with DMARDs and corticosteroids at baseline and within 3 months of ILD diagnosis were similar between the groups (Table 3). The multivariate logistic regression showed that persistently lower albumin level at the 3rd month of follow-up after ILD diagnosis was associated with progression (O.R. =0.02, 95% C.I. 0.00-0.97, p = 0.048) (Table 4).

**Table 2:** Autoimmune and inflammatory markers at the 3rd month of interstitial lung disease diagnosis

	Non-PF-ILD (n = 34)	PF-ILD (n = 34)	p value
**At the 3rd month**			
ESR (mm/hr)	25.0 (19.00-30.50)	33.0 (20.0–48.0)	0.184
CRP (mg/dL)	0.12 (0.04–0.50)	0.32 (0.09–0.84)	0.118
Alb (g/dL)	4.20 (3.83–4.38)	3.85 (3.58–4.13)	0.025 *
IgG (mg/dL)	1525 (1100-1872.5)	1520 (1280–2250)	0.587
**Change between baseline and the 3rd month**			
ESR (mm/hr)	-9.38% (-52.84%-26.39%)	0% (-47.17%-7.27%)	0.916
CRP (mg/dL)	-70% (-92.08%- -10.05%)	-35.93% (-70.0%-3.57%)	0.175
Alb (g/dL)	1.19% (-1.70- 11.94%)	0.00% (0.00-4.65%)	0.626
IgG (mg/dL)	-14.30% (-22.1%-0.0%)	0% (-11.23%-8.58%)	0.026 *

Data are presented as mean ± SD or median (interquartile range), unless otherwise stated

Abbreviations: Alb, albumin; CRP, C-reactive protein; ESR, erythrocyte sedimentation rate; IgG, immunoglobulin G; PF-ILD, progressive fibrosing interstitial lung disease

* p < 0.05

**Table 3:** Treatments received by patients in the cohort at baseline and within 3 months of diagnosis

	Non-PF-ILD (n = 34)	PF-ILD (n = 34)	p value
**Treatments at baseline**			
DMARDs	13 (38.2%)	11 (32.4%)	0.800
Corticosteroids	5 (14.7%)	4 (11.8%)	1.000
Corticosteroids average dosage (prednisolone equivalent dose, mg/day)	10 (8.75–11.25)	11.25 (6.05–22.50)	0.699
Steroid pulse therapy	0 (0.0%)	1 (1.5%)	1.000
**Treatments within 3 months of diagnosis**			
DMARDs use	29 (85.3%)	24 (70.6%)	0.242
Corticosteroids use	24 (70.6%)	21 (61.8%)	0.609
Corticosteroids average dosage (prednisolone equivalent dose, mg/day)	14.65 (10-19.83)	20 (10.94–23.15)	0.151
Steroid pulse therapy	3 (4.4%)	6 (8.8%)	0.476

Data are presented as n (column %), mean ± SD or median (interquartile range), unless otherwise stated

Abbreviations: DMARDs, disease-modifying antirheumatic drugs; PF-ILD, progressive fibrosis interstitial lung disease

**Table 4:** Results of pulmonary function tests after 12-month and 24-month follow-up

	Non-PF-ILD (n = 34)	PF-ILD (n = 31)	p value
**Pulmonary function tests at the 12th month**			
FVC of predicted (%)	90.0 (77.5 to 99.2)	80.3 (53.1 to 92.2)	0.110
DLCO of predicted (%)	59.2 (54.6 to 75.3)	39.3 (30.5 to 65.3)	0.051
cDLCO of predicted (%)	62.5 (58.6 to 73.3)	48.4 (31.2 to 68.8)	0.291
**Pulmonary function tests at the 24th month**			
FVC of predicted (%)	85.3 (7.5.0 to 97.3)	85.3 (72.5 to 98.1)	0.568
DLCO of predicted (%)	60.5 (51.7 to 73.9)	57.0 (38.8 to 74.0)	0.214
cDLCO of predicted (%)	58.8 (53.3 to 68.5)	57.6 (40.2 to 71.0)	0.755
**Relative change between baseline and the 12th month**			
FVC of predicted (%)	11.2 (0.6 to 36.1)	2.5 (-12.1 to 10.6)	0.013 *
DLCO of predicted (%)	9.7 (1.9 to 28.5)	-13.6 (-21.7 to -2.3)	< 0.001 *
cDLCO of predicted (%)	21.8 (0.0 to 55.3)	-20.5 (-23.5 to *)	0.033 *
**Absolute change between baseline and the 12th month**			
FVC of predicted (%)	7.7 (0.6 to 27.4)	1.6 (-1.0 to 7.2)	0.013 *
DLCO of predicted (%)	6.6 (1.1 to 10.6)	-5.1 (-18.4 to -0.6)	0.001 *
cDLCO of predicted (%)	10.6 (0.0 to 22.6)	-18.0 (-20.6 to 4.0)	0.033 *
**Relative change between baseline and the 24th month**			
FVC of predicted (%)	14.2 (2.9 to 29.5)	-1.4 (-11.6 to 12.4)	0.004 *
DLCO of predicted (%)	8.8 (-11.1 to 22.8)	-3.5 (-19.4 to 8.2)	0.099
cDLCO of predicted (%)	0.2 (0.0 to 0.3)	-0.1 (-0.4 to 0.0)	0.035 *
**Absolute change between baseline and the 24th month**			
FVC of predicted (%)	9.0 (2.6 to 17.9)	-1.1 (-7.8 to 9.8)	0.024 *
DLCO of predicted (%)	5.3 (-7.9 to 10.1)	-1.3 (-12.0 to 3.4)	0.108
cDLCO of predicted (%)	11.7 (1.5 to 15.8)	-7.2 (-19.9 to -2.1)	0.035 *

Data are presented as mean ± SD or median (interquartile range), unless otherwise stated

Abbreviations: DLCO, diffusion capacity of carbon monoxide; DLCO/VA, diffusion capacity divided by alveolar volume; cDLCO, diffusion capacity of carbon monoxide corrected for hemoglobin; FCV, forced vital capacity; FEV1, forced expiratory volume in 1 s; PF-ILD, progressive fibrosis-interstitial lung disease

* p < 0.05

In pulmonary function test, non-PF-ILD patients had improvements in both absolute and relative changes of FVC and DLCO, while PF-ILD patients had decline in both FCV and DLCO at the 24th month of follow-up (Table 5). PF-ILD group experienced less increase in FVC (non-PF-ILD vs. PF-ILD: 11.24% vs. 2.54%, p = 0.013) and more decrease in diffusion capacity of carbon monoxide (DLCO) percentage predicted (non-PF-ILD vs. PF-ILD: 9.68% vs. -13.63%, p < 0.001) significantly at the 12th month of follow-up (Table 5). Regarding radiologic presentation, a higher proportion of patients with PF-ILD exhibited honeycombing on HRCT compared to those with non-PF-ILD (non-PF-ILD vs. PF-ILD: 33% vs. 81%, p = 0.030). Furthermore, the total fibrosis score decreased in the non-PF-ILD group during the follow-up (Table 6).

**Table 5:** Characteristics of follow-up high-resolution computed tomography

	Non-PF-ILD (n = 34)	PF-ILD (n = 31)	p value
Total fibrosis score	27.5 (19.6 to 31.3)	44.2 (17.9 to 54.2)	0.164
Honeycombing	3 (33%)	17 (81%)	0.030 *
Reticulation	9 (100%)	21 (100%)	-
Traction bronchiectasis	9 (100%)	18 (85.7%)	0.534
GGO + Traction bronchiectasis	7 (77.8%)	13 (61.9%)	0.675
Consolidation	1 (11.1%)	2 (66.7%)	1.000
Cyst	1 (11.1%)	6 (28.6%)	0.393
UIP	6 (66.7%)	13 (61.9%)	1.000
NSIP	3 (33.3%)	4 (10.0%)	0.640
LIP	0 (0%)	5 (23.8%)	0.286
OP	0 (0%)	0 (0%)	-
**Fibrosis extent compared with baseline**			
Total fibrosis score	-19.6 (-36.3 to -6.9)	0.0 (-17.5 to 11.7)	0.002 *

Data are presented as n (column %), mean ± SD or median (interquartile range), unless otherwise stated

Abbreviations: GGO: ground glass opacity; LIP: lymphocytic interstitial pneumonia; NSIP: nonspecific interstitial pneumonia; OP: organizing pneumonia; PF-ILD: progressive fibrosis-interstitial lung disease; UIP: usual interstitial pneumonia

**Table 6:** Risk factors for PF-ILD assessed using logistic regression analysis

	Univariate Logistic Regression			Multivariate Logistic Regression		
	Odds Ratio	95% C.I.	p value	Odds Ratio	95% C.I.	p value
Age	1.02	0.99–1.06	0.252	-	-	-
Male	1.00	0.38–2.63	1.00	-	-	-
Albumin level at 3rd month	0.09	0.01–0.89	0.039 *	0.02	0.00-0.97	0.048 *
DMARDs use during first 3 months of ILD diagnosis	0.41	0.12–1.38	0.150	-	-	-
Corticosteroids use during first 3 months of ILD diagnosis	1.49	0.54–4.08	0.443	-	-	-
IgG level change during first 3 months of ILD diagnosis	1.04	1.00-1.08	0.044 *	1.00	0.94–1.07	0.985

Abbreviations: Alb, albumin; C.I., confidence interval; DMARDs, disease-modifying antirheumatic drugs; IgG, immunoglobulin G; PF-ILD, progressive fibrosing interstitial lung disease

* p < 0.05

In total of 68 pSjS-ILD patients, the overall survival at 3 years was 91.2%. Kaplan–Meier survival analysis revealed PF-ILD group had significantly poorer 3-year overall survival than non-PF-ILD group (non-PF-ILD vs. PF-ILD: 100% vs. 82.4%, p = 0.011) (Fig. 2). Among female patients, a poor survival outcome was also observed in the PF-ILD group (Fig. 2). In this cohort, there were 6 deaths in the PF-ILD group and all of them were died of respiratory causes. 3-year all-cause mortality were 0% and 17.6% in non-PF-ILD and PF-ILD groups, respectively (p = 0.025).

## Discussion

According to the results of our study, persistently lower albumin levels in the 3rd month of follow-up after ILD diagnosis may be associated with PF-ILD in patients with pSjS-ILD. Disease progression was defined as a two-year period according to the INBULD trial [6]; however, it appeared that an abnormal pulmonary function test could be detected as early as one year after the diagnosis of ILD. Besides, CT honeycombing became more prevalent in PF-ILD patients during 24 months after ILD diagnosis. We also found that the 3-year survival rate in the PF-ILD group was significantly lower than that in the non-PF-ILD group with statistical difference.

Persistently low albumin levels may imply ongoing inflammation. Once the tissues are injured, granulocytes and monocytes are activated and release acute-phase cytokines, such as interleukin-6 [IL-6], which induce the synthesis of acute-phase proteins and simultaneously inhibit the synthesis of albumin [14]. IL-6 promotes lung fibroblast proliferation through a positive autocrine feedback loop [15, 16], leading to persistent inflammation and fibrosis. Some studies reported that low serum albumin levels are associated with increased mortality in IPF [17, 18]. Thus, persistently lower albumin levels may be surrogates of ongoing inflammation and fibroblastic activity in ILDs.

Our study demonstrated non-PF-ILD patients experienced improvements in both absolute and relative changes of FVC and DLCO, while PF-ILD patients experienced decline in both FCV and DLCO at the 24th month. We also observed the similar trends in change of FVC and DLCO at the 12th month. With these findings, we aimed to raise awareness among clinical physicians, emphasizing the importance of close monitoring of PFT to early detect PF-ILD. Besides, some studies also reported changes in DLCO corrected for hemoglobin (cDLCO) are consistent and strong predictors of mortality in patients with various fibrotic lung diseases [19, 20]. Nevertheless, the diagnostic criteria in INBULD trial did not include the parameters of cDLCO. Therefore, hemoglobin testing was not performed routinely at the 12th month and the 24th month after ILD diagnosis in this study, resulting in nearly half of the patients lacking cDLCO data at the 12th month and the 24th month PFTs.

Regarding radiologic findings, we observed a higher prevalence of honeycombing in the PF-ILD group. Adegunsoye et al., conducted an observational study to determine the prevalence and prognostic value of CT honeycombing across diverse ILD subtypes in a multicenter cohort [21]. They found that honeycombing was indicative of a PF-ILD phenotype regardless of underlying diagnosis and had prognostic value in non-IPF ILDs. Notably, usual interstitial pneumonitis (UIP) pattern on HRCT was also associated with worse prognosis in autoimmune ILDs [22, 23]. In our study, among six deceased patients, four of them showed honeycombing and UIP pattern on HRCT.

In our study, there was no significant difference in the proportion of patients receiving DMARDs after ILD diagnosis between the two groups. Few of our patients received anti-fibrotic agents within a year after ILD diagnosis due to a regulatory issue in our country (only 2.9% in non-PF-ILD, 8.8% in PF-ILD, and 5.9% in cohort). Some clinical trials have revealed that the use of anti-fibrotic drugs in PF-ILD may attenuate disease progression, as measured by the decline in FVC [6, 24]. Close monitoring and early anti-fibrotic treatment might benefit these cases.

Our data showed the 3-year overall survival in pSjS-ILD patients was 91.2%, which is consistent with the results of a Chinese retrospective cohort [25]. In our study, we found that PF-ILD patients had poorer survival outcomes, which was consistent with previous literature reports [1, 4, 26]. The impact of sex differences on the prognosis of IPF has been reported in some literature [27, 28]. However, this phenomenon has not been observed in patients with PF-ILD [26] or pSjS-ILD [29, 30]. Limited literature has reported sex discrepancies in PF-ILD patients with primary SjS-ILD. In our cohort, a total of six patients (four females and two males) with PF-ILD died. Among them, we observed poorer survival in females with PF-ILD. However, we were uncertain whether gender differences truly impacted the prognosis of PF-ILD or it was just bias resulted from the limited patient number. Further studies with larger sample sizes are warranted to investigate the impact of sex differences on the prognosis of PF-ILD in patients with primary SjS-ILD.

Our study has some limitations. First, we cannot ensure that all pSS-ILD patients were enrolled because we used ICD as a screening tool, and the participants were recruited from only one tertiary medical center. The number of participants included in this study was small, which may have resulted in an overestimation of the prevalence of PPF in patients with pSjS-ILD. Second, given the retrospective design of our study, there was a presence of missing data, which might result in misclassification and ambiguities in the causative relationship. Third, the prognostic factors for mortality could not be identified because of the small sample size. Fourth, we excluded rapidly progressive ILD, which led to an underestimation of the effect of PPF on mortality in pSjS-ILD. Last but not least, only one participant underwent lung transplantation for PF-ILD during the 3-year follow-up period in this study. Therefore, the impact of lung transplantation on clinical outcomes and survival benefit could not be fully elucidated in our study.

In conclusion, PF-ILD in pSjS-ILD had poorer 3-year survival outcomes. Persistent lower albumin level was associated with PF-ILD. In lung function, PF-ILD patients experienced less increment in both FCV and DLCO at the 12th month and the 24th month. Close monitoring inflammatory markers and PFTs could be helpful for the early detection of PF-ILD. We also found the honeycombing was more prevalent in PF-ILD group on HRCT. Further studies with larger sample size or prospective design are warranted to increase the statistical power.

## Data Availability

All data generated or analyzed during this study are included in this published article.
